# Electron microscopy studies of the coronavirus ribonucleoprotein complex

**DOI:** 10.1007/s13238-016-0352-8

**Published:** 2017-01-02

**Authors:** Miao Gui, Xin Liu, Deyin Guo, Zhen Zhang, Chang-Cheng Yin, Yu Chen, Ye Xiang

**Affiliations:** Department of Biophysics, School of Basic Medical Sciences, Peking University Health Science Centre, 100191, Beijing, China; State Key Laboratory of Virology, College of life Science, Wuhan University, 430072, Wuhan, China; State Key Laboratory of Virology, College of life Science, Wuhan University, 430072, Wuhan, China; State Key Laboratory of Virology, College of life Science, Wuhan University, 430072, Wuhan, China; Department of Biophysics, School of Basic Medical Sciences, Peking University Health Science Centre, 100191, Beijing, China; State Key Laboratory of Virology, College of life Science, Wuhan University, 430072, Wuhan, China; Center for Global Health and Infectious Diseases, Collaborative Innovation Center for Diagnosis and Treatment of Infectious Diseases, Beijing Advanced Innovation Center for Structural Biology, Department of Basic Medical Sciences, School of Medicine, Tsinghua University, 100084, Beijing, China

**Keywords:** Respiratory Syncytial Virus, Infectious Bronchitis Virus, Severe Acute Respiratory Syndrome, Severe Acute Respiratory Syndrome Coronavirus, Murine Hepatitis Virus

**Dear Editor,**

Coronaviruses are enveloped viruses that cause different diseases in humans and animals (Su et al., 2016). Murine hepatitis virus (MHV) causes hepatitis, enteritis and central nervous system diseases in rodents and is one of the best-studied coronaviruses. MHV belongs to the genera betacoronavirus. Members from the same genera also include highly pathogenic coronaviruses such as the severe acute respiratory syndrome coronavirus (SARS-CoV) and the Middle-East respiratory syndrome coronavirus (MERS-CoV) (Vijay and Perlman, 2016).

The coronavirus has a single strand, positive sense RNA genome of about 30 kb, which encodes 4–5 structural proteins, including the nucleocapsid (N) protein, the matrix (M) protein, the small envelope (E) protein, the spike (S) glycoprotein and for some betacoronaviruses, the hemagglutinin esterase (HE) protein (Su et al., 2016). The N proteins bind the viral RNA genome and play important roles in packaging and stabilizing the virus genome, in viral particle assembly and envelope formation, and in the genomic RNA synthesis (McBride et al., 2014). Moreover, it was reported that coronavirus nucleoprotein can regulate host cell cycle, cell stress response, and influence the immune system and other cellular responses (Lu et al., 2011; McBride et al., 2014; Cui et al., 2015; Chang et al., 2016). The N proteins of different coronaviruses are homologous and can be divided into five parts and domains: the N terminal flexible arm, the N terminal domain (NTD), the middle disordered region (LKR), the C terminal domain (CTD), the C terminal flexible tail. The N terminal arm, C terminal tail and the LKR are flexible (Chang et al., 2014). The NTD structures of MHV, SARS-CoV, infectious bronchitis virus (IBV), human coronavirus strain OC43 (HCoV OC43) and the CTD structures of MHV, SARS-CoV and IBV were determined using either x-ray crystallography or NMR (Chang et al., 2016). The determined NTD or CTD structures are highly similar among different coronaviruses. Both the NTD and CTD are shown to interact with the genome RNA while the CTD is also responsible for the dimerization of the nucleoproteins (Chang et al., 2014). The domain crystal structures have provided useful information on the assembly of the ribonucleoprotein complex (RNP), but a lack of the full-length N protein structure and the RNP structure limits our understanding to the assembly and function of coronavirus RNP.

Previous analysis of the RNP extracted from the virus by using negative staining electron microscopy showed that coronavirus RNP might be a long helix with a diameter between 9 nm to 16 nm (Macneughton and Davies, 1978). In this study, we isolated the RNPs from MHV and performed negative staining EM and cryo-EM images analysis of the isolated intact and degraded RNPs. We found that the isolated RNPs are in either relaxed helical sausage-like or supercoiled flower-like structures. Interestingly, we also found that the isolated intact RNPs degraded into small pothook-like subunits. These small subunits could be the building blocks of the long loose helical and the supercoiled flower-like RNP structures.

We performed both negative staining EM and cryo-EM analysis of the MHV (strain MHV-A59) particles. Negative staining images of the intact MHV particles showed that most viral particles had a round shape while some distorted particles were also observed (Fig. 1A). Cryo-EM image analysis of the same sample showed almost all round shaped particles (Fig. 1B), indicating that the distortion in the negative staining images might be caused by the staining procedure. The corona-like spikes around the envelope could be identified in both the negative staining images (Fig. 1A) and cryo-EM images (Fig. 1B). The cryo-EM MHV particles were picked and subjected for 2D classification analyzes. The results showed that the particles have a diameter of ~80 nm to 90 nm (Fig. 1C), which is consistent with the previous EM results (Neuman et al., 2006; Barcena et al., 2009). A dense interior core corresponding to the intertwined RNP is encapsulated inside the envelope (Fig. 1C).

**Figure 1:**
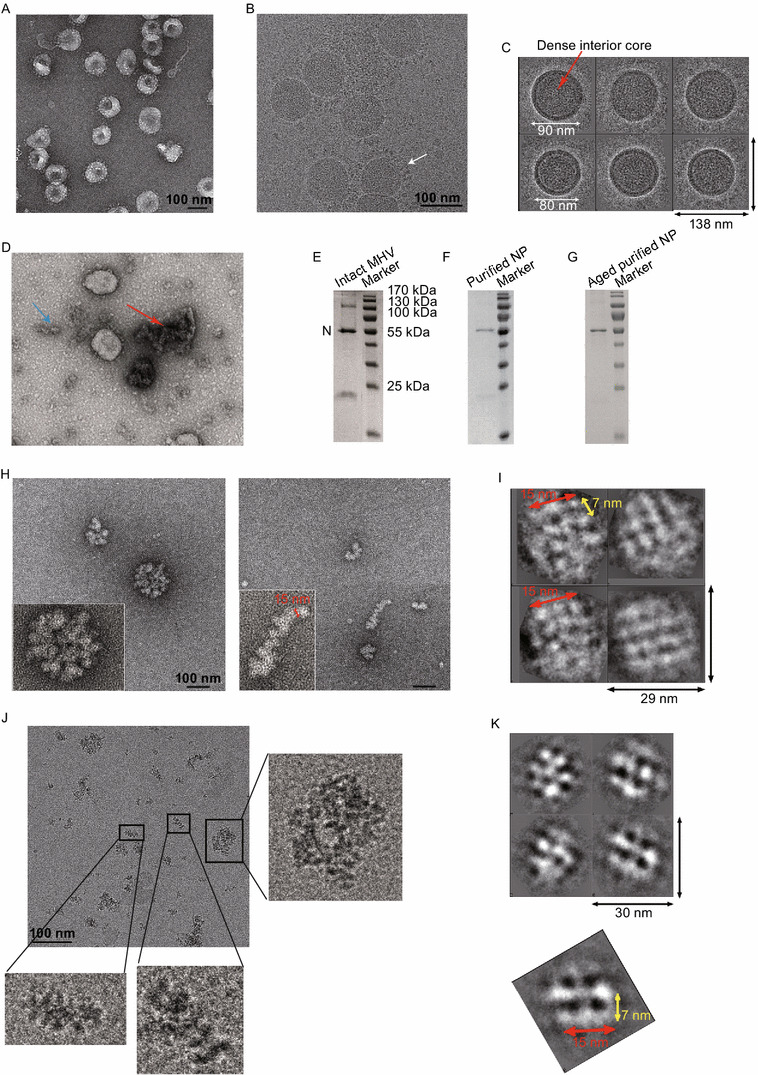
**Electron microscopy study of the RNPs of MHV**. (A) A representative negative staining image of the intact MHV. (B) A representative cryo-EM image of the intact MHV. The white arrows indicated the corona-like spike proteins on the envelope in (B). (C) 2D averaged cryo-EM images of the boxed MHV particles. The virus is round shaped with a dense interior core (red arrow). The diameter of the MHV particles is 80~90 nm. (D) Broken MHV particles after detergent treatment. The RNPs (red arrows) are released. The blue arrow indicates possible RNP fragments. (E) SDS-PAGE gel analysis of the intact MHV. The band corresponding to the N protein is marked (confirmed by mass spectrometry analysis). (F) SDS-PAGE gel analysis of the purified MHV RNPs. (G) SDS-PAGE gel analysis of the purified aged MHV RNPs (at 4°C for about one week). (H) Negative staining images of the fresh purified MHV RNPs. Typical RNPs are zoomed in and shown at the left bottom of each image. (I) 2D averaged negative staining images of the fresh purified MHV RNPs. Particles boxed along similar helical RNP filaments as shown in (H). (J) Cryo-EM images of the fresh purified MHV RNP. The RNPs have different shapes. Some RNPs are compact intertwined assemblies (right) while some RNPs are helical filaments (bottom). (K) 2D averaged cryo-EM images of the fresh purified MHV RNPs. Particles boxed along similar helical RNP filaments as shown in (J)

We then broke the MHV particles by incubating the particles in a lysis buffer containing ~3% CHAPS. Negative staining analysis showed that most of the virus particles were broken and the RNPs were released after the treatment. The released RNPs are in either a loose filament structure or in a compact flower-like assembly that may be similar to the intact RNP assembly in virus particles (Fig. 1D). There were also some smaller particles, which might be the RNP fragments (Fig. 1D).

SDS-PAGE gel analysis of the intact virus showed that the N protein is about 55 kDa (Fig. 1E). To investigate the structural details of the released RNP, we further purified the RNPs from the broken MHV by using a sucrose cushion. After the purification, the N protein is the only visible band on the Coomassie brilliant blue stained SDS-PAGE gel (Fig. 1F).

Further negative staining and cryo-EM analysis of the purified RNPs showed two major morphologies of the RNPs, including the compact intertwined flower shape and the loose filament shape structures (Fig. 1H and 1J). Image analysis showed that the diameter of the filaments is approximately 15 nm (Fig. 1H). Particles boxed along the filaments were subjected for 2D classification analyzes. The 2D averaged images showed lattice-like patterns, which have distinct strands with a distance of about 7 nm between the adjacent strands (Fig. 1I and 1K). The repeating strands packing along the long axis of the loose filament structure suggest possible helical arrangements of the N proteins, which are similar to these of some other viral RNP structures (e.g., the influenza virus, respiratory syncytial virus (RSV)) (Zhou et al., 2013).

To investigate the stability of the extracted MHV RNPs, we placed the purified RNPs at 4 degree for about one week. Interestingly, electron microscopy image analysis showed that the purified aged RNPs are in a complete different morphology. Most of the large RNP assemblies disappeared, leaving only small particles in a relatively uniform size (Figs. 2A, S1A and S1B). The negative staining images were subjected for further processing. 2D classification analysis showed that these particles have a size of about 7 nm × 7 nm (Fig. 2B). 3D reconstructions calculated using both EMAN2 and RELION1.4 from the boxed images showed a twisted pothook-shape structure (Figs. 2C and S1). Overall the reconstructed density displays pseudo-two fold symmetric features except for the densities at the two distal ends of the pothook (Fig. 2C). The diameter of the 3D density map (~7 nm) is similar to the distance between the adjacent strands of the lattice-like pattern along the long RNP filament (Fig. 1I and 1H). The length of two subunits (14–15 nm) is close to the width (~15 nm) of the helical RNP filament (Fig. 1I and 1H). The crystal structure of the CTD of MHV N protein and biochemical assays showed that the N proteins form a dimer (Chang et al., 2016). SDS-PAGE gel analysis of our aged RNPs showed that the N proteins are intact and have little degradation (Fig. 1G), indicating that the N proteins would still be in a stable oligomerized state that is resistant to protease degradation. The volume of the reconstructed subunit density is enough for accommodating two N protein molecules, suggesting that the subunit likely contains an N dimer. We then fitted the NTD (PDB accession number: 3HD4) and CTD (PDB accession number: 2CJR) crystal structures of the N protein into the 3D density map. The CTD dimer structure was placed and fitted at the pseudo-two fold symmetric central part of the pothook, whereas two NTD monomers were fitted at the two asymmetric arms of the pothook (Fig. 2D). The remaining un-interpreted extra densities may belong to the N terminal arm, the LKR and the C terminal tail of the N protein and RNA fragments (Fig. S2). Our interpretation of the subunit structure is consistent with previous SAXS analysis of the SARS N protein dimers in solution, in which the NTDs also adopt asymmetric conformations (Chang et al., 2009).

**Figure 2:**
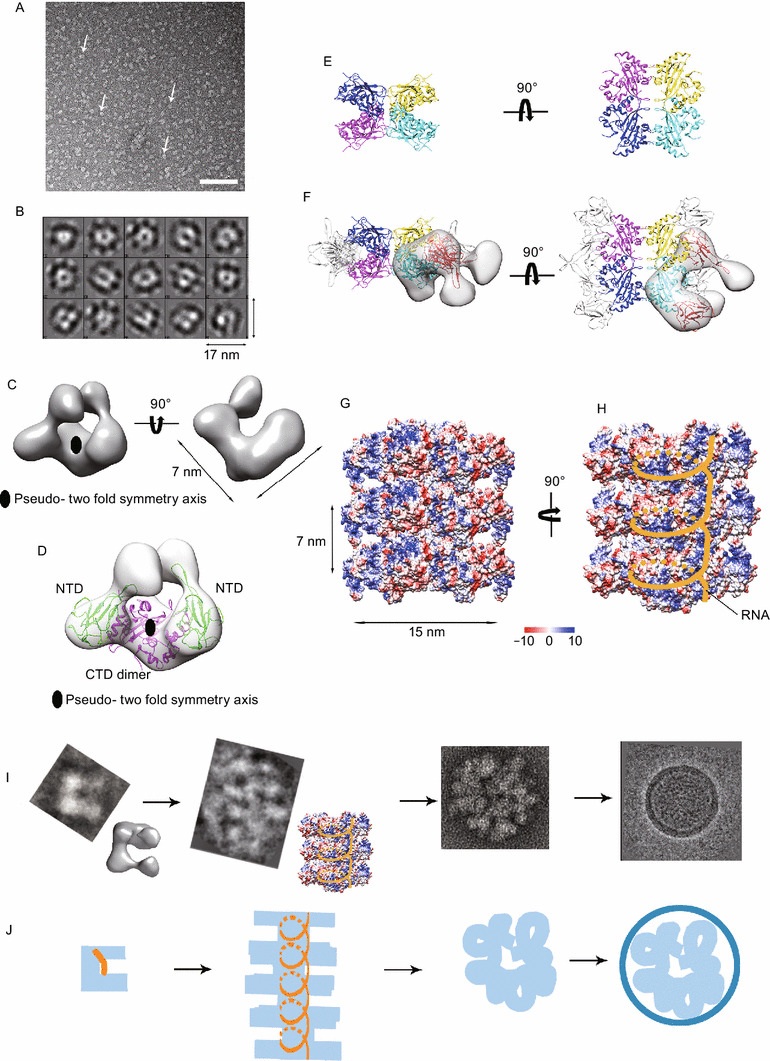
**3D reconstruction of the purified aged MHV RNPs and a model of the MHV RNP assembly**. (A–C) A representative raw negative staining image (A), 2D averaged images (B) and 3D reconstructions (C) of the purified aged MHV RNPs. White arrows in (A) indicate RNP particles. Scale bar, 100 nm. (D) Docking of the crystal structures of the MHV NP NTD monomer (PDB accession: 3HD4) and the SARS-CoV NP CTD dimer (PDB accession: 2CJR) into the 3D density map. The NTDs are colored green. The CTDs are colored magenta. (E) Two CTDs package to form an octamer in an asymmetry unit of the crystal. Four CTD dimers (colored blue, yellow, cyan and magenta, respectively) form an “X” shape. (F) Superimpositions of four N protein dimers (built in (D)) onto the CTD octamer as shown in (E). Density map of one RNP subunit is shown in gray semi-transparent surface with the corresponding fitted ribbon models colored cyan (CTDs) and red (NTDs). NTDs of other three N protein dimers are colored gray. (G) A RNP fragment model built based on the model proposed in (F). Three octamers stack along the helical axis. Surface rendered diagram of the RNP fragment model is shown. (H) Surface rendered diagram of the RNP fragment model showing possible RNA binding grooves (indicated by orange lines). Positively charged regions of the surface are colored blue. Scale bar, −10 to 10 Kcal/(mol·e-). (I) Schematic diagrams showing the assembly of the RNPs. The N proteins assemble to form pothook-shape building blocks, which then intertwine with genome RNA and package to form a helical structure. The helical structure may then supercoil to form a flower-like RNP core packaged in the viral envelope. (J) Cartoon diagrams showing a possible assembling procedure of the RNPs. The genome RNA is shown in orange lines or dash lines

Crystal structural studies of the CTDs showed that the CTDs pack head to head into octamers (Fig. 2E) and form a twin helix in the crystal (Chang et al., 2014). A putative model of the RNP was proposed based on the crystal packing of the CTDs. Based on our fitting result, we superimposed subunit densities onto the crystal CTD octamers and generated a model of the RNP (Fig. 2F and 2G). In the model we proposed, two subunits pack head to head into a helical filament. The width of the modeled filament is twice of the subunit (~15 nm). Distance between the repeating strands, which is the distance of the two arms of the pothook, is around 7 nm. The parameters of the modeled filament are consistent with our observation for the isolated RNP filament. The coronavirus N protein interact with RNA at multiple regions, including CTD, NTD and the LKR region (Chang et al., 2014). The residues at the positive charged groove of NTD are reported to be crucial for the N protein and RNA interaction in coronavirus (Chang et al., 2016). Based on these biochemical data and the surface electrostatic potential of the proposed model, we identified possible RNA binding sites of the N protein and proposed possible genomic RNA binding groove in the helical RNP model (Figs. 2H and S2C).

Cryo-EM tomography studies of the intact MHV have found that the helical RNP is extensively twisted upon itself in the envelope (Fig. S3) (Barcena et al., 2009). The cryo-tomo result is consistent with our observation of the isolated RNPs, most of which are supercoiled into a compact intertwined structure. However, a small portion of the isolated RNPs was also in a loose helical filament form, suggesting that the RNPs become unstable upon losing the interaction with the transmembrane M protein and the envelope (Risco et al., 1998; Barcena et al., 2009). Degradation of the genome RNA might be mediated by trace RNase contaminations, which truncate the long RNP yielding short sausage-like, solenoid-like and the pothook-like fragments.

The coronavirus has a large genome of about 30 kb, while the overall size of the viral particle is comparable with that of other RNA virus (for coronavirus, ~85 nm in diameter; for human immunodeficiency virus, 120–170 nm in diameter, ~2 × 9 kb genome size (Briggs et al., 2003); for human respiratory syncytial virus, 150–250 nm in diameter, ~15 kb genome size (Mejias and Ramilo, 2015)). It seems that the space inside the envelope would be inadequate for a coronavirus to encapsulate loosely packed RNPs. In a way similar to the eukaryotic cell genome packaging, coronaviruses package the genome to form a supercoiled dense structure (Fig. 2I and 2J) for their large genome packaging. Sequence alignments of the N proteins showed more than 24% identity among different coronaviruses (Fig. S4) and structural comparison of the coronavirus N proteins also showed high similarity. Thus the pothook shape subunit we observed could be a common building unit of all the coronaviruses for the assembly of their RNPs. Taking together, our results here provide new insight into our understanding of the coronavirus RNP assembly.

## Electronic supplementary material

The online version of this article (doi:10.1007/s13238-016-0352-8) contains supplementary material, which is available to authorized users.

## Supplementary Material

13238_2016_352_MOESM1_ESM MATERIALS AND METHODS

## Footnotes

We thank the Tsinghua University Branch of the National Center for Protein Sciences (Beijing) for providing EM and Computing facility support. We are grateful to Xiaomin Li and Jianlin Lei for their help in cryo-EM tomography data collection. This work was supported by the National Basic Research Program (973 Program) (No. 2015CB910100), the National Key Plan for Scientific Research and Development of China (2016YFA0501100), the National Natural Science Foundation of China (Grant Nos. 31470721 and 81550001) and the Beijing Advanced Innovation Center for Structural Biology.

Y.X., Y.C. and M.G. designed the project. Y.C. Z.Z. and X.L. purified the MHV virus. M.G. performed the EM and other experiments. M.G., Y.X. analyzed the data and wrote the initial manuscript. All authors contributed on editing the paper.

Miao Gui, Xin Liu, Deyin Guo, Zhen Zhang, Chang-Cheng Yin, Yu Chen, Ye Xiang declare that they have no conflict of interest. This article does not contain any studies with human or animal subjects performed by the any of the authors.
