# Supplementary material for: Electron microscopy studies of the coronavirus ribonucleoprotein complex
Source: Protein Cell. 2017 Jan 2;8(3):219–24. doi: 10.1007/s13238-016-0352-8 (PMC5326623; doi:10.1007/s13238-016-0352-8)
Supplement: 13238_2016_352_MOESM1_ESM — MATERIALS AND METHODS [file 13238_2016_352_moesm1_esm.pdf]

METERIALS AND METHODS

MHV preparation

MHV-A59 (NC_001846) viral particles were collected from infected Neuro-2a cell line as described (Cui et al., 2015; Wang et al., 2015). The cell culture was centrifuged at 13,000 rpm for 30 min at 4 ℃ to remove the cell debris and the supernatant was collected and centrifuged again at 32,000 rpm for 2 h at 4 ℃ to pellet the virus. The precipitant was resuspended with 1 ml Tris-NaCl buffer (20 mM Tris-HCl at pH 8.0 and 100 mM NaCl). The resuspended virus (1 ml) was loaded onto a 10%~60% (w/v) step sucrose gradient (total 9.6 ml, 1.6 mL/layer) and centrifuged at 22,000 rpm for 20 h at 4 ℃. Viral particles were collected from the interface between 20% and 30% sucrose layers. The collection was diluted with the Tris-NaCl buffer. The virus was pelleted by centrifugation at 35,000 rpm for 2 h at 4 ℃ and was then resuspended with 100 μl Tris-NaCl buffer. The purified MHV viral particles were stored at 4 ℃.

RNP purification and SDS-PAGE gel analysis

The MHV particles were lysed by incubating the sample in a buffer containing ~3% CHAPS (Sigma-aldrich co. llc usa), 20 mM HEPES-Na at pH 7.6 and 150 mM NaCl for 30 min at 4 degree. The lysate was then loaded on a 20% sucrose cushion prepared in a buffer containing 20mM HEPES-Na at pH 7.6 and 150 mM NaCl (HEPES buffer). The RNPs were pelleted by centrifugation for 30 min at 21,130 × g. To remove the sucrose, the pallet was washed twice with the HEPES buffer by

centrifugation for 5 min at 21,130 × g. The aged RNPs were prepared by placing the purified RNPs at 4 degree for about one week. The intact MHV particles, fresh purified and aged RNPs were analyzed using 12% SDS-PAGE gels and EM.

Negative staining EM analysis of the intact MHV virus and the purified RNPs

For negative staining specimen preparation, 4 microliters of samples were applied onto a glow discharged continuous carbon grid (Beijing XinxingBraim Technology Co., Ltd.). After waiting for approximate 1 minute to let the grid adsorb enough materials, excess sample was blotted away from the grid with filter paper. The grid was immediately washed twice with 1% uranyl acetate (UA) solution and was then incubated with the UA solution for additional 1 min. The UA solution was completely removed with filter paper and the grids were air-dried at room temperature.

For cryo-EM specimen preparation, 3 microliters of samples were applied onto a glow discharged continuous carbon grid with a layer of continuous ultrathin carbon film (TED PELLA, INC.). Each grid was blotted for 9-11 s, plunged into liquid ethane using a Gatan Cryoplunge 3 System.

Cryo-EM specimens and the negative staining specimens of the purified aged RNP were examined under an FEI Tecnai F20 electron microscope equipped with an FEG filament. The microscope was operating at 200-kV acceleration voltage, using a nominal magnification of 62,000 × (pixel size: 0.135 nm). Negative staining specimens of the fresh purified RNPs were examined under an FEI Tecnai Spirit Bio-Twin electron microscope equipped with a LaB_6 filament operating at 120 kV

acceleration voltage. All images were recorded using Gatan 895 4 k × 4 k CCD cameras with an exposure dose of 20-30 $e^{-}/Å^{2}$. The defocus range used was -1 μm to -3.5 μm.

Cryo-EM tomographic tilt series were collected with an FEI Titan Krios microscope equipped with a Falcon II camera at a pixel size of 0.29 nm. The FEI tomography software was used for the data collection. Each tilt series covered an angular range from -64° to 64° with a tilt step of 2°. The defocus range used was -4 um to -6 um. The total dose was about 110 $e^{-}/Å^{2}$ for each tilt series.

Image Processing

For the negative staining images of the purified aged RNP, 24,826 particles from 55 micrographs were boxed using the EMAN2 program e2boxer.py (Tang et al., 2007). The initial model was generated by the EMAN2 python script e2initialmodel.py and low-pass filtered to 60Å. Reference-free 2D class averages were performed by using EMAN2 and RELION1.4 (Scheres, 2012). Particles of bad class averages were discarded and 22,060 good particles were used for the 3D auto-refinement. To validate the accuracy of the reconstructions, we did the refinement and reconstruction with both EMAN2 and RELION1.4 using the same low-pass filtered initial model and same particle dataset. Both 3D refinements were performed following a "gold standard" procedure, wherein the raw data were randomly divided into two subsets that were then refined independently. The resolution was 25 Å at Fourier shell correlation value 0.5 and 19 Å at Fourier shell correlation value 0.143. Docking of the

crystal structures into 3D reconstruction map was performed in UCSF Chimera (Pettersen et al., 2004).

Particle boxing and reference-free 2D class average analyzes of the fresh purified RNPs were performed using the EMAN2 software package. 3D density maps were visualized using UCSF Chimera.

The tomographic data were processed with the software Protomo (Noble and Stagg, 2015). The tilt series images were aligned for several iterations and then the aligned images were back-projected to generate the 3D reconstructions. The tomograms were denoised by band-pass filtering and virtualized using the software IMOD (Kremer et al., 1996).

Tilt-pair Validation

For tilt-pair validation experiment, data were collected at 0 °and 20 °tilt angles for each region of the grid, using an FEI Tecnai Spirit Bio-Twin electron microscope equipped with a LaB_6 filament and operated at 120-kV acceleration voltage at a pixel size of 0.16 nm. A total of 39 particle pairs were used for the validation test. The EMAN2 reconstruction density map was scaled to the same pixel size and the same boxsize as those of the tilt-pair images before it was used as the validation reference. The tilt-pair validation test was performed using the EMAN2 python script e2tiltvalidate.py. About 17 of the 39 particle pairs were inside the red cycle around the corresponding angle (~44%).

RERERENCES

Kremer JR, Mastronarde DN, and McIntosh JR (1996) Computer visualization of three-dimensional image data using IMOD. J Struct Biol 116:71-76

Larkin MA, Blackshields G, Brown NP, Chenna R, McGettigan PA, McWilliam H, Valentin F, Wallace IM, Wilm A, Lopez R, et al. (2007) Clustal W and Clustal X version 2.0. Bioinformatics 23:2947-2948

Noble AJ, and Stagg SM (2015) Automated batch fiducial-less tilt-series alignment in Appion using Protomo. J Struct Biol 192:270-278

Pettersen EF, Goddard TD, Huang CC, Couch GS, Greenblatt DM, Meng EC, and Ferrin TE (2004) UCSF Chimera--a visualization system for exploratory research and analysis. J Comput Chem 25:1605-1612

Robert X, and Gouet P (2014) Deciphering key features in protein structures with the new ENDscript server. Nucleic Acids Res 42:W320-324

Scheres SH (2012) RELION: implementation of a Bayesian approach to cryo-EM structure determination. J Struct Biol 180:519-530

Tang G, Peng L, Baldwin PR, Mann DS, Jiang W, Rees I, and Ludtke SJ (2007) EMAN2: an extensible image processing suite for electron microscopy. J Struct Biol 157:38-46

Wang Y, Sun Y, Wu A, Xu S, Pan R, Zeng C, Jin X, Ge X, Shi Z, Ahola T, et al. (2015) Coronavirus nsp10/nsp16 Methyltransferase Can Be Targeted by nsp10-Derived Peptide In Vitro and In Vivo To Reduce Replication and Pathogenesis. J Virol 89:8416-8427

SUPPLEMENTARY FIGURES

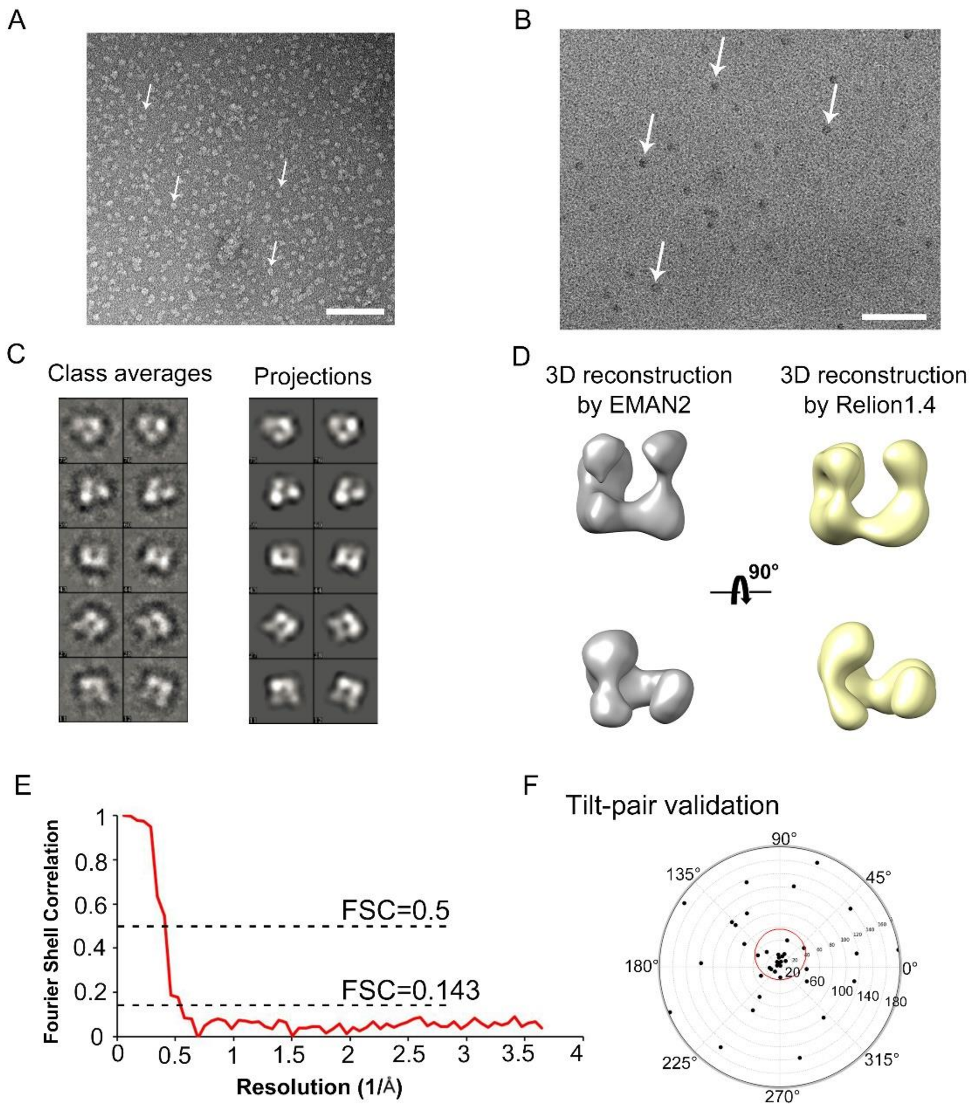

Supplementary figure S1. Image processing and validation of the purified aged RNPs. (A) A representative raw negative staining image of the purified aged MHV RNP particles. (B) A representative low-pass filtered Cryo-EM image of the purified aged MHV RNP particles. Scale bar, 100 nm. (C) Representative 2D class averaged images and the corresponding projection images of the 3D reconstruction map. The 2D class averaged images are consistent with the corresponding projections, suggesting that the 3D reconstruction is reliable. (D) 3D reconstruction maps

calculated by using EMAN2 and RELION1.4. (E) Fourier shell correlation curves of the 3D reconstruction. The resolution was 25 Å at FSC value 0.5 and 19 Å at FSC value 0.143. (F) Tilt-pair validation result. The red circle denotes particle pairs that cluster around the experimental tilt geometry. ~44% particle pairs were inside the red cycle.

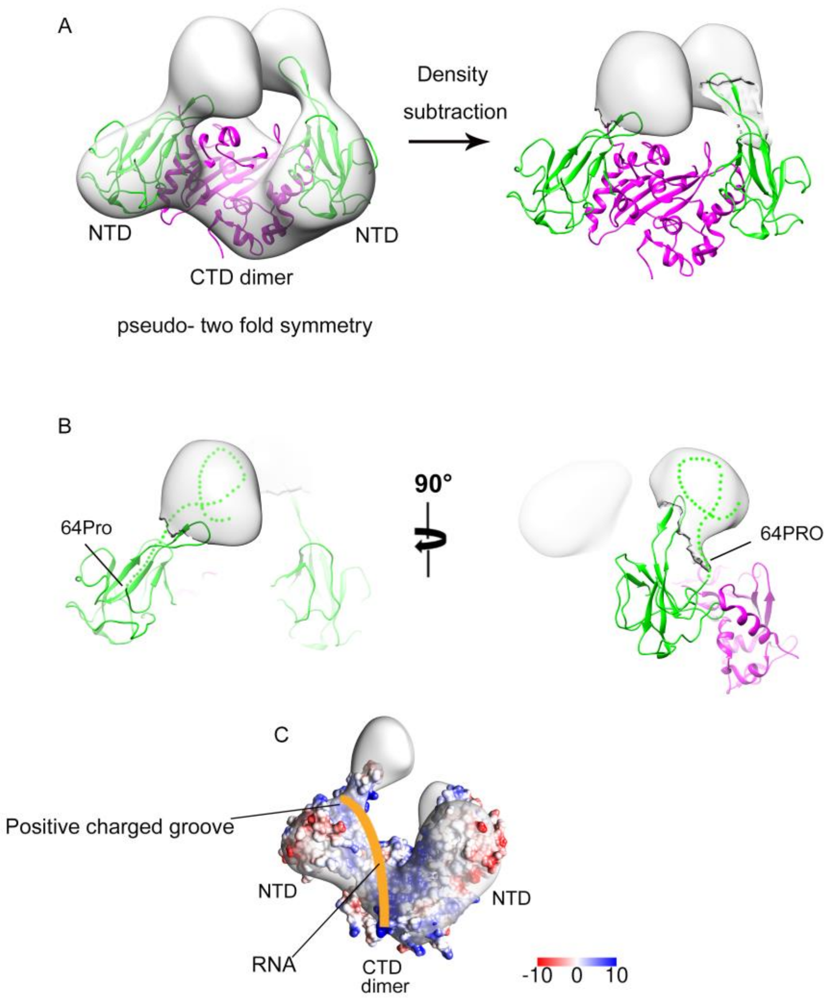

Supplementary figure S2. Interpretation of the 3D density map. (A) A difference map calculated between the 3D density map and the fitted models showing the un-interpreted densities. Densities within 2 Å of the fitted models were set to zero in the 3D density map. (B) Two views showing the un-interpreted bulky densities at the two distal ends of the 3D density map. These extra densities are close to the N terminal (64Pro) of the NTD domains and may be the density of the missing N-terminal 63 amino acids (modeled using dotted lines). (C) Possible RNA binding site with one N protein dimer. Model of the N protein dimer is presented as electrostatic potential surface. Positive, neutral and negative charges are colored blue,

white and red. 3D density map is shown in transparent gray. RNA is represented with orange lines. Scale bar, -10 to 10 Kcal/(mol.e-).

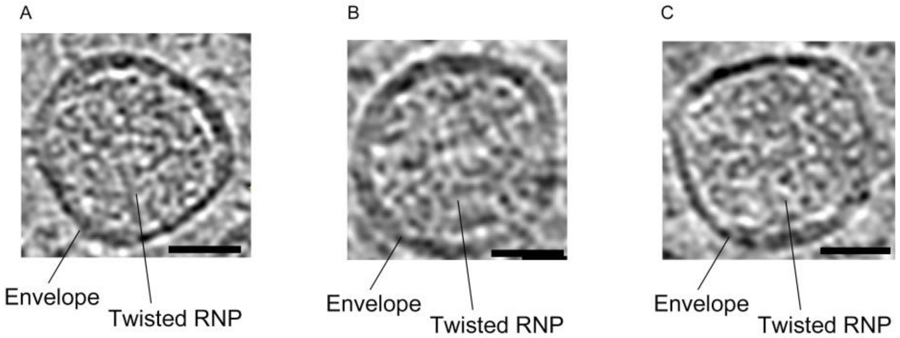

Supplementary figure S3. Cryo tomography of MHV particles. (A)-(C) Tomographic sections of the 3D reconstructions of MHV particles. The twisted RNPs and the envelope surrounding the RNPs were indicated. Scale bar, 50 nm.

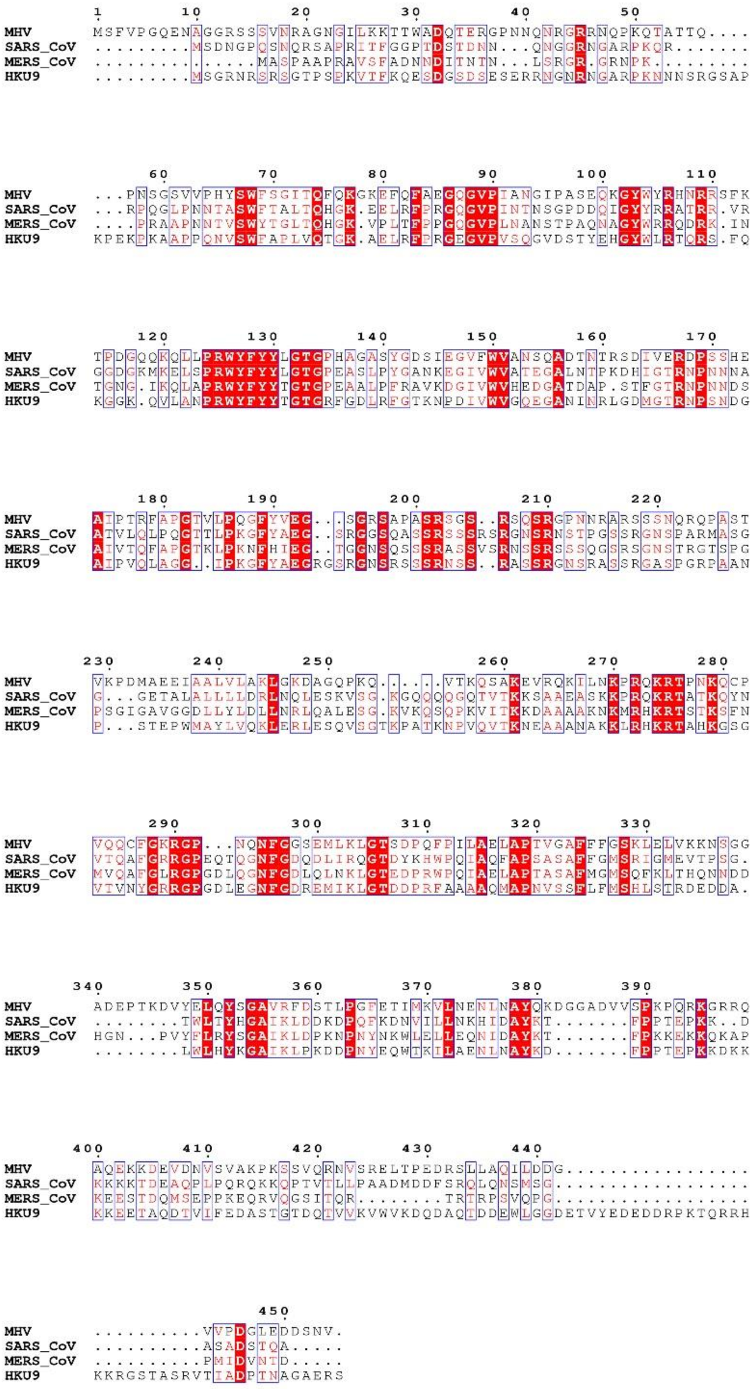

Supplementary figure S4. Sequence alignment of the N proteins of different coronavirus. Sequence alignments of MHV (Group A of betacoronaviruses), SARS-CoV (Group B), MERS-COV (group C) and HKU9 (group D) N proteins. The alignment was performed with the program ClustalX (Larkin et al., 2007). Identical

residues and similar residues are shown in blue boxes and identical residues are highlighted as red. This figure was created with the program ESPript (Robert and Gouet, 2014).
